# Dr. Horner on the Mucous Membranes

**Published:** 1828-04-01

**Authors:** 


### Medico-Chirurgical Review,

### No. XVI.

[FASCICULUS IV.]

FEBRUARY 23, 1828.

### Art. X.

*An Inquiry into the healthy and diseased Appearances of the Mucous Membrane of the Stomach and Intestines.* By W. E. Horner, M. D. Professor of Anatomy in the University of Pennsylvania.

[American Journal of the Medical Sciences, No. 1.]

THE disputes which have obtained respecting the natural and the morbid appearances of the gastro-intestinal mucous membrane, sufficiently attest the great importance of settling these mooted points by some acknowledged standard. Like Dr. Horner, we have frequently seen the most opposite conclusions drawn from identical appearances in the organs under consideration, without having any available experience or authority, by which the correctness of these conclusions could be determined.* These circumstances induced Dr. Horner to institute a series of observations on the mucous membrane of the stomach and bowels; and, by coincidences purely accidental, he was so fortunate as to obtain that description of information, in a few months, which, in the ordinary current of events, might not have been acquired in years.

"Three unsettled points present themselves in this inquiry:—1st. What is the healthy condition and appearance of the gastro-intestinal mucous membrane? 2d. What is its appearance in congestion from the agonies of dying? 3d. What is its appearance in genuine red inflammation?"

So much ambiguity and vagueness have obtained respecting the meaning of certain terms, especially inflammation and congestion, that our author thinks it necessary to state that, "by congestion, he means an accumulation of red blood in any part or organ of the body, without irritation or mechanical violence; and, by red inflammation, the accumulation of red blood which follows any local irritation."

"1. *Of the Natural Colour of Mucous Membranes.*

"When an animal is bled to death, the stomach being empty, the mucous mem-

* See our review of Mr. Annesley's work on the Effects of Calomel on the Mucous Membrane, in the 4th volume of this Series, p. 333, *et seq.*

brane of the latter, and of the intestines, is of a yellowish pearl colour, presenting at a short distance the lightest possible tint of pink; and few or no marks of blood exist, even in the large vessels under the peritoneal coat.

"*Experiment* 1*st. April*, 1827.—At a butchery in Spring Garden, a sheep, which had fasted for twenty-four hours, was slaughtered in the usual way, by cutting the throat, and immediately afterwards dividing the spinal marrow in front between the occiput and first vertebra. As soon as life was extinguished we examined the abdomen. The internal membrane of the stomach was of a pearl colour, slightly yellow, approaching to what artists call a light tint of bright brown, and the intestines were almost exactly like the stomach. Neither stomach nor intestines exhibited their blood-vessels, even of the largest size, except very faintly, and there were no red patches in either.

"*Experiment* 2*d*.—Was a repetition of the first experiment, and was followed by precisely similar results.*

"If an animal be killed with a full stomach by puncture of the medulla spinalis at its commencement, the mucous coat of the stomach will retain on the surface where the food was in contact with it, a light lake, approaching vermilion, from the detention of blood in its capillary system. The following experiments proved this :—

"*Experiment* 3*d. June* 1827, at the University. A full grown female rabbit of the white kind was fed upon green cabbage leaves. In about three hours afterwards she was killed by pithing with a saddler's awl between the occiput and the first vertebra. Immediately upon the introduction of the instrument, the body was seized with a spasm, the extremities were drawn together and convulsed; the animal gaped two or three times, but there was no inspiration. In five minutes after pithing, the abdomen was opened; the circulation was found by pressure upon the veins to be going on but languidly in the parietes of the abdomen and in the abdominal viscera. I waited five minutes more, when it seemed to be completely stopped.

"I then cut out the stomach, laid it open along its great curvature and washed away with a stream of water its contents, which presented the appearance of having been well boiled and bruised. The food would have measured probably about an ounce, and was principally in the left half of the stomach: there was no hour-glass contraction of the latter. The mucous coat of the left half of the stomach was of a uniform light lake, approaching vermilion, without blotches or shades of difference, and firm; but the mucous coat of the right half was almost destitute of vessels or injection, and had the dull pearl colour of the stomachs of the sheep bled to death. The mucous coat of the small intestines was the same colour as the stomach, but much lighter.

"The vermicular motion continued for ten or fifteen minutes in the intestines, after the abdomen was opened. I observed that the same occurred in the horns and body of the uterus, quite as clearly as in the intestines, and that it could be produced at pleasure by puncture, by clipping, and by stirring. On the uterus being slit open from one end to the other, it flattened itself out, and the internal surface corrugated precisely as in the small intestine. The capillary system of the liver bled freely half an hour after death, upon being punctured with the scissors.

"*Experiment* 4*th*.—A full grown female rabbit—Opened the abdomen, by a cut from sternum to pubes—intestines became very highly injected with blood, in a few minutes after being exposed. Laid open the colon and irritated the internal coat by rubbing—it took on a bright scarlet lake colour, approaching to vermilion; —on the external face of the same intestine, an anastomosis was manifested resembling a very fine honey-comb. The stomach was laid open by a section along its greater curvature, and the bouillie-like contents consisting of green vegetable matter turned out. The internal coat of the stomach highly suffused with blood,

---

* "These two experiments, or rather observations, were executed by Dr. La Roche and myself in commencement of a joint series, from which, much to my regret, his departure for Europe withdrew him at this point of our inquiry."

and of a deep crimson lake colour. The stomach bled very profusely from the cut surface, in consequence of which, it began to clear up, by its colour becoming less intense. Applied some alcoholic solution of corrosive sublimate upon one spot of the internal coat of the stomach, about ten minutes after the commencement of the experiment—it did not seem to produce any impression, perhaps owing to the hæmorrhage. Killed the animal in twenty-five minutes after commencement, by pithing it behind occiput; then cut out the stomach. The cardiac half was of a light tint of lake colour; the pyloric half was of a yellow pearl colour. The stomach after death, in this experiment, corresponded with experiment No. 3, in the colour being uniform, not mottled. The peristaltic movements were observed in the uterus and its cornua. After pithing, gasping occurred, with the usual cessation of respiration.

"*Experiment 5th.*—A full grown rabbit. Punctured medulla spinalis, at commencement, respiration stopped immediately. In five minutes opened abdomen, capillary circulation as well as that in the larger vessels, brisk. In twenty minutes capillaries of liver, spleen, and kidnies bled on being punctured with a saddler's awl—stomach and bowels did not. Cut the stomach open along its greater curvature and turned out its contents, then removed and washed it well immediately. The left half of a lake, approaching vermilion, uniformly diffused as in experiments 3d and 4th, the right half of a dull pearl, as in the same experiments."

Dr. Horner observes that, "from the great number of blood-vessels distributed through mucous membranes, they are, during life, of a very bright red colour, on many of the viscera, as the stomach, the small, and the lower end of the large intestines, on the vagina and nose." In many other parts, they are much less vascular, as in the lining membrane of the sinuses of the nose, in the bladder, and in the excretory ducts generally. "The nostril and the vagina, in a robust healthy person, will probably be found to represent correctly the shade of colour which, in life, belongs naturally to the gastro-intestinal mucous coat."

"*Observation 1st. Living Colour of healthy Mucous Coat of Colon.*—There is now a female in the Surgical Ward of the Philadelphia Alms-House, who suffered some time ago from prolapsus ani, which is said to have protruded about six inches · the protruded intestine sloughed off, as well as the sphincter ani and the adjoining integuments. This new state of the parts affords a distinct view of the internal coat of the colon, near the sigmoid flexure. The perineum has cicatrized and united to the end of the colon, but the surface is kept excoriated, by the continued excrementitious discharges; from the want of a sphincter. In this patient, the mucous coat is of the colour of the vagina, or of a recently blistered surface of the true skin."

The second observation (in the dead body) adduced is, that of a man who died suddenly, (after 20 minutes' illness,) and was examined 12 hours after death. The cause of death was disease of the heart, but this we need not detail. Some parts of the internal surface of the stomach (summits and sides of the rugæ) were of a brown, some of a pearl colour. The same was the case in the jejunum; but the inner surface of the colon was of a dull pearl colour.

Dr. Horner never saw abdominal viscera more healthy than in this case, and, therefore, thinks the appearances presented may be taken as a standard.

### II. COLOUR OF MUCOUS MEMBRANE IN CONGESTION.

Dr. Horner confesses, that our materials for elucidating the laws and phenomena of passive congestion are exceedingly imperfect. He thinks, however, we may fairly infer, that the congestion of red blood in any part of the body is commonly produced by an obstruction of one or more of the large venous trunks which return the blood to the heart. This is exemplified by ligature on the arm, tight garters, or other article of dress, tumours pressing on the course of vessels, &c. But then, in such cases, the congestion disappears the moment the obstruction is removed, and the function of the part is restored. If, however, the congestion continues an undue length of time, the congested part will inflame, or even mortify, as in strangulated hernia. The following case is curious in many points of view, besides bearing on the physiological, or rather pathological, point in question.

*Case.* W. Thompson, aged 37, was admitted into the Philadelphia Almshouse, on the 18th June, 1827, with abscess around the cricoid cartilage, and died two days afterwards.

" *Previous History.*—While in France, some years since, had secondary syphilis and other constitutional affections,—was salivated for a length of time—recovered—came to this country; where he has continued since in the capacity of coachman. Has drank freely for some time past. About four months since, had common catarrh, the result of which was some difficulty in deglutition and respiration, and tremor in the throat.

" *Symptoms.*—When admitted, there was a flattened, immoveable tumour in front of the thyroid cartilage, about two inches in diameter, half an inch thick—inspiration difficult in the extreme, amounting almost to suffocation—expectoration purulent—deglutition exceedingly painful—countenance anxious and distorted.

" The tumour was removed by the knife on the day of admission, at four o'clock, P.M. I found it lying on the whole front part of the thyroid cartilage, between it and the sterno-hyoid muscles. The wound was filled with lint, and then covered with a compress of the same, maintained by a roller: in ten or fifteen minutes afterwards, it began to bleed rather freely. I then removed the dressings, turned the clot of blood out of it, sponged, and not finding any bleeding vessel, I directed it to be left undressed, with a light cloth thrown over it. This answered to arrest the bleeding until midnight, when it again bled half a pint and then stopped. The tumour was of the hard encephaloid kind, having, however, a small purulent softening in front; it seemed as if it might have come from a lymphatic gland; but its flattened shape was adverse to this idea, neither had it a distinct capsule.

" He spent the night but slightly relieved by the operation, pulse rather full, and tense—expectoration and deglutition less painful and difficult—the former thick, white, purulent and consistent—Ordered venesection ℥viij. ℞. Tart. Ant. gr. ij.—Aq. pura, ℥viij. M. A table spoonful every hour—took six doses, then stopped on account of nausea.

" *June 9th.*—Respiration very laborious—expectoration as yesterday—will not consent to tracheotomy, which I now proposed to him. On the tenth, died at ten o'clock, A.M.

" *Autopsy, six hours after death.* An entire removal of the tumour over the thyroid cartilage—its bed thickly covered with coagulating lymph—thyroid gland healthy—superior laryngeal nerves not affected—No marks of capillary congestion, on the surface of the body—Lungs healthy in structure, some old pleuritic adhesions on left side—no particular congestion, save at their posterior part, perhaps less blood than natural found on cutting into them—miliary deposits of coagulating lymph on the surface of both lungs."

In the pericardium there was a small quantity of fluid. The system of the vena portarum was filled with blood, even in the fine intestinal branches, giving a purple colour to the alimentary canal. The colon externally was of a pearl colour. There was the mark of an abscess in the liver. The peritoneal surface of the stomach was pearl-coloured—the internal surface of a light pink generally—summits of rugæ like red streaks, studded with minute dots of red. The mucous membrane of the small intestines was of similar appearance, and the inner lining of the colon presented a tinge of red.

"*Throat.* Along the upper margin of the glottis, as formed by the doubling of the membrane from the tip of the arytenoid to the side of epiglottis cartilage, a tumour existed on each side, formed of serum and coagulable lymph, about the size of a small nutmeg, so loose as to hang over the glottis, and to be drawn over the rima glottidis in every act of inspiration.

"Cricoid cartilage, at posterior part both externally and internally, separated from its perichondrium; whose surface was in the condition of a fistulous sore.

"Aryteno-cricoid articulation, detached by the extension of the latter disease. A sinus formed communicating between the fistula and the right ventricle of the larynx. Posterior part of cricoid cartilage reduced to a thin edge above,—its diseased surface, rough and resembling a carious bone—lining membrane of trachea and bronchia, natural."

There was some serous effusion, and marks of congestion in the head.

To sum up on this head, it would appear, 1st. That congestion is not an active condition of the part affected. 2dly, That congestion most frequently is the result of mechanical impediments to the venous circulation. 3dly, That the other cases in which it occurs, are, where there is a want of reciprocal sympathy between the blood and the blood-vessels in the capillary system, in consequence of which, the latter refuses passage to the red blood.

We now come to an important division of this interesting subject.

### III.—OF THE RED INFLAMMATION OF MUCOUS MEMBRANES.

Dr. Horner, after making some general observations on the difference between inflammation and congestion, proceeds thus:—

"The traces of acute inflammation are in many cases very fugitive, and entirely disappear upon death, because the local irritation which attracted the blood and accumulated it, having ceased, the blood abandons that part and retires towards the centre of the circulation. We can seldom tell by the appearances twenty-four hours after death, the quantity of blood which has penetrated an inflamed membrane, as the peritoneum, the pleura, the cellular and mucous membranes, the skin, &c. The eruption of measles, and the redness of sore throat disappear on the death of the patient. We are not, however, to infer from these circumstances, that the mere afflux of blood to a part constitutes inflammation, on the contrary it is attended with a dilated condition of the vessels independent of this afflux, for if death occur during the height of measles, the eruptions may be made to reappear by injecting the vessels.

"Bichat* has very properly observed, that in inflammation we should distinguish between acute and chronic affections, because, though the blood readily vanishes from the former, yet it will remain in the latter, because it has combined with the diseased organ. Hence, induration, suppuration, and vitiated secretions are satisfactory signs of inflammation of a part.

"The increased irritability of a part is the cause of its inflammation, and of the afflux of fluids to it. Frequently this increased irritability depends upon the sudden diminution of irritability in other parts of the body by depressing applications. Thus, cold suddenly applied to the surface by depressing the irritability there, causes an accumulation of it with inflammation of some one of the internal organs, as the lungs or bowels. Direct undue stimulation of an organ or part, will produce an augmentation of its irritability amounting to inflammation. The natural increase of irritability of organs at particular periods of life, sometimes amounts to inflammation, as that of the uterus at puberty manifested by menorrhagia, amenorrhœa, &c. the extreme tenderness of the breast at the beginning of lactation—the tenderness of the testicles during a state of sexual excitement, &c.

"It is principally in the capillary system,† that the phenomena of inflammation occur, and that the varying degrees of organic sensibility determine corresponding movements in the circulating fluids, accumulating them at one spot, and expelling them from another. Inflammation is therefore exactly the inverse of what BOERHAAVE believed, for, according to him, the blood pushed from behind by the heart into an organ caused its irritation or inflammation; whereas it is the irritation which attracts the blood.

"Irritation has all the gradations from the light, alternating, and varying blush of virgin diffidence, to that rapid and tumultuous afflux of fluids to an organ which in a few hours produces its dissolution. In slight irritations there is no fever, but when they are more intense there is an increased heat of the body, and an accelerated circulation throughout it. Fever in such cases is merely a general symptomatic affection, arising from the sympathy which connects the heart to all other parts, and has nothing of the specific affection in it, but merely manifests the grade of the irritation. Thus, a fever from a syphilitic bubo is the same as a fever from measles, from small pox, or even from a mechanical injury."

Dr. H. remarks that, in acute inflammation of the mucous membrane of the stomach, when the patient dies in the early stage of the disease, the blood-vessels which ramify through the stomach, are enlarged and distended with blood. The membrane itself is covered by a coat of mucus, which is sometimes limpid, like the white of an egg—but on other occasions, thick and purulent, like that from the nose in the last stage of catarrh.

"The mucus adheres frequently very strongly to the stomach, and is now and then so tenacious and consistent from the admixture of coagulating lymph with it, as to resemble a false membrane. When the coat of mucus is scraped and washed away, the mucous membrane itself is brought into view, being most frequently in the greater part of its extent of a deep red, approaching on some occasions, a crimson red, on others, a purple or black. These colours are owing to the injection of a prodigious number of capillary vessels in the mucous coat; and in addition to them, we find the inflamed part of the stomach interspersed with bands and patches of red,

* "Anat. Gen. vol. 2, p. 22." † "Idem, p. 25."

of the colour of coagulated blood, being a species of ecchymosis, in which the blood has escaped beneath, and in the substance of the mucous membrane. The mucous membrane at these places is somewhat softer than natural, and sometimes appears swollen, may be readily detached from the cellular coat with the end of a scalpel, and is in the condition of a bouillie. In cutting through all the coats of the stomach, and looking at the incised edge, it will be seen that where the general and diffused redness exists, the colour is only superficial; but at the ecchymosed spots, not only the whole thickness of the mucous coat is concerned, but even the corresponding part of the muscular is more highly coloured than in common. In some cases where the gastritis has been occasioned by caustics, there are eschars of the mucous membrane, some of which are detached and leave the muscular, or even the peritoneal coat bare from the depth of the impression made. It is said that such eschars become more distinct some hours after the stomach has been exposed to the air.*

"In some cases of acute inflammation where the symptoms have been those of army dysentery, or of typhus fever, the stomach and bowels are occasionally found a little thicker than usual, and of a yellowish brown or red colour on their peritoneal surface, and in their thickness; the fœtor from them on opening the abdomen, is much greater than usual, and the peculiar smell of the halitus from the peritoneum is overcome by it.

"Some circumstances have been alluded to, by Drs. PHYSICK and CATHRALL,† which, if properly appreciated, would contribute much to settle the opinions of medical men in relation to gastric inflammation; for they go to show that the appearance of the inflammation of yellow fever varies according to the date of the disease. 'In two persons who died of this disease on the fifth day, the villous membrane of the stomach, especially about its smaller end, was found highly inflamed, and this inflammation extended through the pylorus into the duodenum some way.' The inflammation was exactly similar to that produced by arsenic.

"'In another person who died on the eighth day of the disease, several spots of extravasation were discovered between the membranes, particularly about the smaller end of the stomach, the inflammation of which had considerably abated. Pus was seen in the beginning of the duodenum, and the villous membrane at this part was thickened.'

"'In two other persons who died at a more advanced period of the disease, the stomach appeared spotted in many places with extravasations, and the inflammation disappeared.' It and the intestines contained a black liquor so acrid as to produce inflammation and swelling on the operator's hands, which continued for some days.

"'The stomach of those who died early in this disease was always contracted; but in those who died at a more advanced period, where extravasations appeared, it was distended with air.' The external surface of the stomach was healthy, but from its veins being distended with blood, they had a dark appearance.

"In the dissections performed by Dr. Physick in the years 1798-99,‡ the inside of the stomach in some cases resembled the black vomit precisely in colour. In most of these cases no black matter was found in the cavity of the stomach, but the blackness depended upon this fluid being retained in the vessels of the inflamed mucous membrane. The doctor remarks that he never observed any putridity attending it, and that the colour was very distinguishable from the dark purple of gangrene. In some stomachs the blackness was universal; in others, in spots only; there being some spots in a state of high inflammation, and giving the inside of the stomach a chequered appearance. These spots, in one instance, resembled one another precisely in shape, and in all other respects, excepting colour, in which they differed, one being black and the other red."

In chronic inflammation of the mucous membrane of the stomach, this tissue

---

* "Dict. des Sc. Med. T. 17."

† "Rush's Inquiries, Vol. 3, p. 172. Philadelphia, 1809."

‡ "Medical Repository, New York, Vol. 5, p. 129."

is commonly found thrown into numerous folds. Sometimes it is thickened—of a denser texture than natural, and reddish, with irregular white patches. On other occasions, the whole of it is red, with purple spots, as in acute gastritis. Sometimes the whole of it is purple, approaching to a claret colour. Poisons of a force sufficient to cause sudden death, leave small ulcers near the pylorus, and along the great curvature of the stomach.

"In some observations that I have made on the stomachs of intemperate persons at the Alms-house, I have found the mucous coat thickened and dense, without any remarkable contraction of the stomach, yet thrown into numerous, thick, elevated rugæ, and the summits of those rugæ, so reddened by numerous capillary vessels injected with blood, that at the distance of a few feet they appeared, when the distinction of the individual capillaries was lost in the distance, like red streaks. This, which is probably one of the pathognomonic signs of a recent debauch in an old drunkard, is frequently mistaken in the post mortem examinations at the Alms-house, for congestion, from an arrest of blood in the agonies of dissolution."

The red blotches which form the leading anatomical character of acute mucous inflammation in malignant fevers, may be readily produced by chemical irritants.

A full grown rabbit, after being fed for three hours on cabbage, had the abdomen opened. The stomach was contracted in the middle, like an hour-glass. The whole mucous coat was of a scarlet lake colour. On wiping or rubbing the inner surface, the colour became deeper. Some solution of sublimate in spirits of wine was applied, and rendered the hue still more intense than before. The mucous membrane of the small intestines presented a light lake colour, which remained after death.

Dr. Horner has introduced a number of cases as well as experiments, illustrative of various morbid states of the gastro-intestinal mucous membrane; but our limits are exceeded, and we must pass them over very cursorily. It was the opinion of Dr. Physic that, in very high grades of gastric irritation, there is neither pain nor vomiting—the state of inflammation being so exalted, that its effects approximate those of the most deleterious poisons, which cause sudden death without local pain, fever, or any very sensible derangement of the functions, except a mere sense of debility and illness. The following is considered an example.

"Henry Turner, a black man, aged fifty-five, assistant in the apothecaries shop, at the Alms-house, after an apparently slight indisposition of a few days, which seemed to require rest rather than any thing else, while sitting up in his bed, suddenly expired almost without a struggle, May 4th, 1827. On dissection, twenty-four hours afterwards, the stomach was found of middle size, thick and dense, its mucous coat thrown into numerous, well-marked, elevated rugæ, and almost universally of a deep arterial red; the red globules of blood were extravasated in numerous spots and blotches in the thickness of the mucous coat and along the summits of the rugæ. This was a patient of Dr. Hodge, and I witnessed the dissection by chance. I declared unhesitatingly that the disease was an exasperated inflammation of the stomach, but owing to the want of an assignable cause for it, as well as the want of appropriate symptoms during life, the opinion was not very readily acquiesced in. In a few days afterwards it was ascertained through Mr. Marks, the apothecary at the Alms-house, that Turner had been taking private draughts from the bottle of Hoffman's Anodyne; at least, the quantity missing, could be accounted for in no other way."

Several cases of children are given, with the view of illustrating the same principle. Thus, a child, 8 months old, was apparently in health, and playing on the lap of its aunt, when it was taken suddenly ill, and died instantly. This occurred after taking a hearty draught of cow's milk, to which the child was accustomed. The body was examined 24 hours after death. There was some turgescence in the cerebral vessels—no disease in the thorax. In the stomach was found about a gill of a cheese-like coagulum of milk. The mucous coat of this organ was not vascular, but of a pearl colour. As small pieces of the said coagulum were found in the duodenum in an indigested state, it is highly probable, that the gastric irritation was the cause of death in this case. Several others are related, where children were seized with convulsions, and suddenly expired, presenting undigested substances in the stomach, and no other circumstance to account for the fatal termination.

"These observations upon the stomachs of children, illustrate sufficiently the fact, that the most extreme irritations of the stomach may exist during life, and may even be fatal, but yet not be manifested after death by unusual redness of the tissues affected. It would be indeed unphilosophical, and inconsistent with pathological observations on other parts, to exact from the stomach an invariable manifestation of disease, by redness and injection of its mucous surface after death. Let the redness of the skin in erysipelas be ever so strong during life, it frequently disappears wholly, by the retreat of the blood from its capillaries during death. Measles are similar in this respect, and the fact already mentioned is very worthy of attention, that if a fine injection be thrown into a subject who has died at the height of the eruption, the vessels originally dilated by the irritation, manifest themselves by the greater quantity of injecting matter they receive ; or in other words, the eruption may be renewed after death, as I have satisfactorily ascertained by experiment. If any conjecture could be hazarded on these points, we are disposed to believe that during the process of death, the vitality of parts in a state of inflammation is frequently so far diminished that they no longer have the power of attracting the fluids in undue quantity to them, consequently their redness disappears.

"Acute inflammation of mucous tissues generally only thickens inconsiderably the part affected. It is attended by an increased secretion of mucus, of serum, of fibrino-mucous matter ; and even an exhalation of red blood, from the blood-vessels being so extremely superficial. We see continually these phenomena going on in inflammation of the Schneiderian membrane and in colitis.

"It has been seen that if an acute inflammation of the gastro-intestinal mucous membrane does not kill in its early stages, the injection with red blood is frequently by no means so great as it would have been in the case of an earlier death, and the changes which I have generally observed, are as follow :—The stomach and bowels assume a dirty yellow or liquorice colour, the stomach presents internally small blotches of red at intervals, and frequently are found small filaments of coagulated blood adhering to the mucous coat of the stomach, seemingly at the orifices of the vessels from which they were discharged. The veins of the bowels are either partially or generally filled with blood. The intestinal mucus is abundant, adheres closely, and is tinged yellow by the bile. The mucous membrane of the stomach is easily peeled or scraped off by the finger nail. The brain in this state most frequently exhibits marks of congestion, and with some inflammation of its meninges. The eyes and skin are yellow. In the skin there are apt to be left blotches of red blood, resembling purpura."

We must now conclude our analysis of Dr. Horner's memoir. It is accompanied by coloured representations of the various conditions of the mucous membranes, executed in a very masterly style. The Essay is extremely creditable to the judgment and talent of our esteemed transatlantic confrère.

